# P-313. Trends in Invasive *Staphylococcus aureus* infections in Monroe County, NY, 2016-2023

**DOI:** 10.1093/ofid/ofae631.516

**Published:** 2025-01-29

**Authors:** Marissa Walsh, Lauren Nakamura, Anita Gellert, Elizabeth Keller, Marissa Tracy, Christina Felsen, Ghinwa Dumyati

**Affiliations:** New York Emerging Infections Program, Rochester, New York; University of Rochester, Fairport, New York; New York Emerging Infections Program, Rochester, New York; New York Emerging Infections Program, Rochester, New York; University of Rochester Medical Center, Rochester, New York; New York Emerging Infections Program, Rochester, New York; New York Emerging Infections Program and University of Rochester Medical Center, Rochester, New York

## Abstract

**Background:**

Since the COVID-19 pandemic, increases in healthcare-associated invasive methicillin-resistant *S. aureus* (MRSA) infections were observed. Limited data exist for invasive methicillin-sensitive *S. aureus* (MSSA) infections. We identified trends in community and healthcare associated invasive *S. aureus* (iSA) infections during 2016-2023.

**Figure 1.**
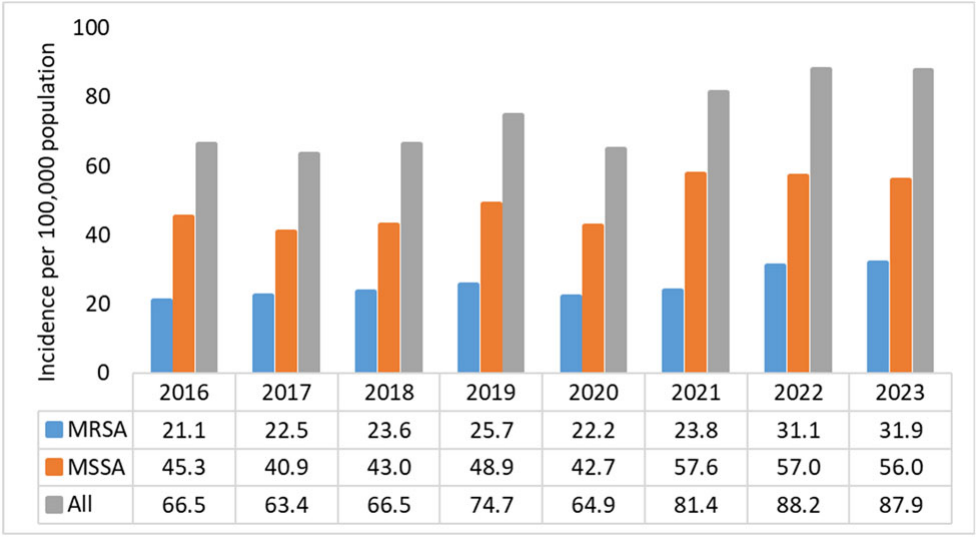
Incidence of iSA infections by resistance-type, Monroe County, NY, 2016-2023.

**Methods:**

Active, population-based surveillance data for iSA was collected as part of the CDC Emerging Infections Program. A case was defined as a resident of Monroe County, NY with *S. aureus* isolated from a normally sterile site. Cases were classified as hospital onset (HO) if the culture was obtained >3 days after hospitalization, healthcare-associated community-onset (HACO) if the culture was obtained in an outpatient setting or < 3 days after hospitalization in a patient with ≥1 prior major healthcare exposures, or otherwise community-associated (CA); incidence was calculated for each epidemiologic classification.

**Figure 2.**
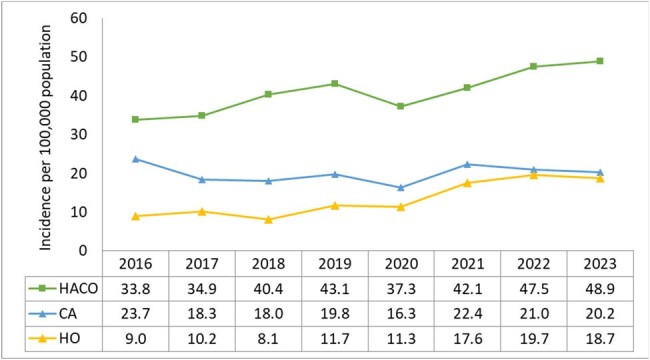
Incidence of iSA infections by epidemiologic classification, Monroe County, NY, 2016-2023.

**Results:**

A total 4,440 iSA cases were identified during 2016-2023; 3,512 had *S. aureus* isolated from blood and 942 were from other sterile sites. iSA incidence increased from 66.5 (per 100,000 population) in 2016 to a peak of 87.9 in 2023 (Figure 1). HACO comprised >50% cases and incidence mirrored the pattern of overall rates, rising from 33.8 in 2016 to 48.9 in 2023 (Figure 2). HO incidence doubled, rising from 9 in 2016 to 18.2 in 2023; CA incidence remained stable.

MSSA incidence was approximately twice the rate of MRSA for each year. Incidence of MSSA rapidly increased from 2020 to 2021 (42.7 to 57.6) with increases in all epidemiologic classes (Figure 3). MRSA rose the following year (23.8 to 31.1) with a decline in CA cases; HO increased by 59% and HACO increased by 33%.

**Figure 3.**
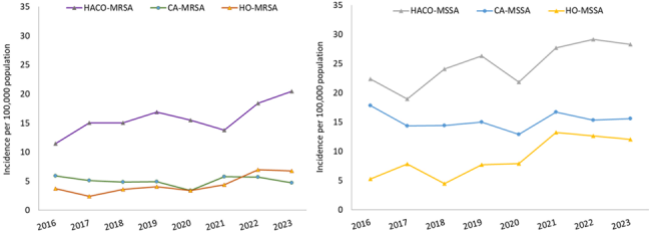
Comparison of epidemiologic classification between MRSA vs. MSSA cases, Monroe County, NY, 2016-2023.

**Conclusion:**

Our study highlights an increase in iSA infections since 2016, predominantly driven by HACO cases. MSSA infections surpassed MRSA infections throughout, with both experiencing sustained increases since COVID-19 emerged. These findings emphasize the importance of continued surveillance and targeted interventions to address the growing burden of iSA infections, particularly those associated with healthcare.

**Disclosures:**

**All Authors**: No reported disclosures

